# Enhancing Vascularized Composite Allograft Supercooling Preservation: A Multifaceted Approach with CPA Optimization, Thermal Tracking, and Stepwise Loading Techniques

**DOI:** 10.21203/rs.3.rs-4431685/v1

**Published:** 2024-06-11

**Authors:** Irina Filz von Reiterdank, Antonia T. Dinicu, Curtis L. Cetrulo, J.H. Coert, Aebele B. Mink van der Molen, Korkut Uygun

**Affiliations:** Massachusetts General Hospital; Massachusetts General Hospital; Massachusetts General Hospital; University Medical Center Utrecht; University Medical Center Utrecht; Massachusetts General Hospital

**Keywords:** Vascularized Composite Allografts, Supercooling, Preservation, Subzero non-freezing, Thermal, FLIR, CPA

## Abstract

Vascularized composite allografts (VCAs) present unique challenges in transplant medicine, owing to their complex structure and vulnerability to ischemic injury. Innovative preservation techniques are crucial for extending the viability of these grafts, from procurement to transplantation. This study addresses these challenges by integrating cryoprotectant agent (CPA) optimization, advanced thermal tracking, and stepwise CPA loading strategies within an ex vivo rodent model. CPA optimization focused on various combinations, identifying those that effectively suppress ice nucleation while mitigating cytotoxicity. Thermal dynamics were monitored using invasive thermocouples and non-invasive FLIR imaging, yielding detailed temperature profiles crucial for managing warm ischemia time and optimizing cooling rates. The efficacy of stepwise CPA loading versus conventional flush protocols demonstrated that stepwise (un)loading significantly improved arterial resistance and weight change outcomes. In summary, this study presents comprehensive advancements in VCA preservation strategies, combining CPA optimization, precise thermal monitoring, and stepwise loading techniques. These findings hold potential implications for refining transplantation protocols and improving graft viability in VCA transplantation.

## INTRODUCTION

**Figure 1:**
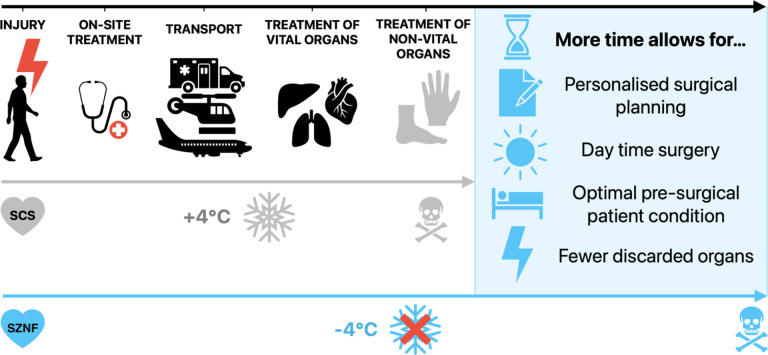
Supercooling Presents a Promising Avenue to Extend Organ Preservation Time. Extending the preservation time of non-vital organs by ‘organ banking’ has the potential to allow for better, personalized surgical planning, daytime surgery, improvement of overall patient condition, and eventually reduction of complication rates, resulting in better patient outcomes and quality of life. *SCS Static Cold Storage; SZNF Subzero Non-Freezing*.

The preservation of vascularized composite allografts (VCAs) poses complex challenges in transplant medicine, primarily due to their sensitivity to ischemic damage and the inherent challenge of storing heterogeneous tissues like skin, muscle, and bone [1, 2]. Optimal storage conditions and minimization of immunological rejection are critical to the success of VCA transplantation. Although evolving methods such as supercooling present promising avenues to extend preservation times, these advancements bring forth their own set of intricate challenges. Subzero non-freezing, or supercooling, has been proposed as a method to reduce the metabolism and thereby allow for the extension of preservation of whole organs (Fig. 1) [3]. Extending organ preservation improves logistics, opportunity for patient matching in a global context, storage of lost organs by military personnel in low resource environments, and importantly, would allow for mixed chimerism protocols which enable immunosuppression-free transplantation but require at least 48h of organ storage for recipient preparation [4]. In clinic, VCA storage is limited to 6h of SCS due to the ischemia susceptibility of muscle tissue. Machine perfusion in rodent transplant studies has shown to enable graft recovery, thereby extending Static Cold Storage (SCS) [1] and warm ischemic preservation time limits [5], however, the maximum storage time was still 12h and 3h, respectively. Using supercooling in livers, storage time increased up to 4 times in rats [6] and up to 27h in humans [7]. Early supercooling studies in VCAs at −1°C have shown intriguing results for extended storage [8], however, no studies were performed at lower temperatures.

Central to the efficacy in organ preservation is the optimization of cryoprotective agents (CPA). In supercooling CPAs suppress ice nucleation at subzero temperatures. However, this must be balanced with its cytotoxicity to the organ. CPA optimization methods for VCAs are limited to conventional ex vivo models, which although comprehensive, present limitations due to their low throughput and complexity. Recent investigations include those on the use of zebrafish for high-throughput CPA assessment for cardiac preservation [9] and of larvae for mosquito preservation [10]. These have shown the efficacy of employing lower concentrations of a broader range of CPAs in enhancing tissue viability while achieving the desired phase, in the case of supercooling by mitigating ice crystallization. However, the variability in results across different systems [11], as seen in contrasting findings between hepatocyte and liver preservation studies [6, 7, 12], underscores the need for more focused CPA research, particularly in the context of VCAs containing ice nucleators such as hair, bone, and nails [13, 14]. This discrepancy along the translational pathway and between organ systems leaves more to be desired on CPA optimization and shows the impact of the system being used. Another critical aspect of CPAs is the management of osmotic shock, which has the potential of being mitigated by techniques applied ex vivo. This method, proven effective in microfluidic devices[15] and in rat liver studies applying partial freezing [16], involves gradually introducing and removing CPAs, thus preventing abrupt changes in osmotic pressure that could damage tissue integrity.

In addition to CPA-related challenges, the intricate interplay between CPA distribution and temperature regulation plays a crucial role in organ preservation. The success of cryopreservation hinges on ensuring adequate perfusion and, thereby, CPA penetration to prevent freezing in all areas of the graft. Assessment of perfusion and CPA penetration requires a sophisticated understanding of temperature gradients within the tissue, which is as important as the CPA optimization itself. Bulk tissues present distinct challenges in heat and mass transfer dynamics during subzero preservation, necessitating thermal tracking methods to reduce unnecessary ischemic time and optimizing cooling and warming rates. Composite grafts, in particular, consist of different tissue types which creates the potential for differences between heat transfer rates dependent on the tissue type. To assess heat transfer, a dual approach of using both invasive and non-invasive methods will be employed to accurately chart temperature fluctuations within the VCA throughout the supercooling and rewarming phases.

This study explores the advancements in CPA optimization and thermal assessment, focusing on the efficacy of stepwise CPA loading and unloading strategies alongside invasive and non-invasive temperature monitoring techniques in an ex vivo rodent VCA model. By addressing these key aspects, we aim to enhance the current practices in VCA preservation and improve transplantation outcomes.

## METHODS

### Animals

22 inbred, male Lewis rats (250 ± 50 grams) were used for all experiments (Charles River Laboratories, Wilmington, MA). The animals received humane care in accordance with the National Research Council guidelines and the experimental protocols were approved by the IACUC of Massachusetts General Hospital (Boston, MA) and the Animal Care and Use Review Office. Authors complied with the ARRIVE guidelines.

Studies are performed in three phases: (1) Determination of CPA solution-based freezing risk with and without tissue (n = 8); (2) Thermal tracking (n = 2); (3) Pressure-controlled, stepwise switches between solutions (n = 12).

### CPA freezing assessment

To test the freezing points of different storage solutions, 15 mL plastic test tubes were filled with 10 mL of the chosen solution and covered with 0.5 mL of paraffin oil[17] prior to placing in a cooler (MHD13, Engel, Jupiter, FL). This method is known as deep supercooling, which uses interface sealing to minimize heterogenous ice nucleation. For convenience, we will simply refer to this protocol as supercooling. Temperature was set to 0°C and lowered by 1°C every 24 hours. Based on visual observation, freezing of the solution in the vials was assessed. To establish a baseline, commonly used clinical storage solutions Histidine-Tryptophan-Ketoglutarate (HTK) (25767–735-45, Essential Pharmaceuticals, Ewing Township, NJ) and University of Wisconsin solution (UW) (MPS-001, Bridge to Life, Elkhorn, WI), and VCA perfusion solution, known as modified Steen (Steen+)[1], were tested (n = 3 vials per solution).

Next, previously established supercooling solution for VCAs [6, 7, 18] was assessed for freezing with varying glycerol concentrations (2–5%) (5516, Sigma Aldrich, Madison, WI) as well as Ethylene glycol (EG) (102466–1L, Honeywell, Dusseldorf, Germany) at 5% v/v concentration. This solution consists of HTK as the base solution, based on a previously established VCA preservation solution (HTK with 35kDa Polyethylene glycol (PEG) 5% (94646–1KG-F, Sigma Aldrich, Madison, WI) + Trehalose 50 mM (J66006.09, Thermo Fischer, Bend OR) + Glycerol 5%) with n = 21–23 vials per condition [18]. EG was tested as a replacement for glycerol due to its lower viscosity and success in livers [16].

**Figure 2:**
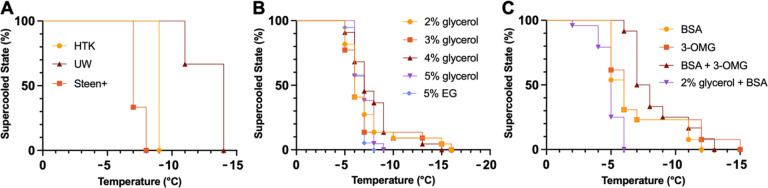
Stability of the Supercooled State of Different Static Solutions. (**A**) Freezing assessment of clinical preservation solutions in vials using visual observation of ice formation shows more effective freezing point depression of UW compared to HTK and Steen+ (n=3 per condition). (**B**) 5% glycerol in the VCA supercooling solution reached the lowest temperatures before freezing. However, these conditions were not statistically significant using the Mantel-Cox test for significance (n=21–23 per condition). (**C**) Additions to HTK had no major differences in freezing temperatures between groups. Nonetheless, the groups with additions showed first signs of freezing at higher temperatures and had extended temperature ranges at which freezing occurred (n=13 per condition).

Finally, the effect of various additions related to osmotic and oncotic regulation were assessed in the supercooling solution (HTK + PEG 5% + Trehalose 50 mM + 5% glycerol). The tested additives were 15% Bovine Serum Albumin (BSA) (A7906–5KG, Sigma Aldrich, Madison, WI), which is a key ingredient of Steen+; 100 mM 3-OMG, which has shown success as an additive to the loading phase in both livers and VCAs; 15% BSA + 100 mM 3-OMG to test the convergent effect. To test the effect of BSA 15% on the lowest possible CPA level to avoid organ toxicity, while maintaining reliable non-freezing at −4°C (Fig. 2B), the addition of BSA 15%was also tested in the supercooling solution with 2% glycerol. All these solutions are tested with n = 13 vials per condition.

As the presence of tissue can influence the freezing point, the effect of the addition of partial and whole hindlimbs on the freezing point was assessed for alternative storage solutions using Steen + as a base solution [1]. Eight hindlimbs, 4 partial [19] and 4 whole [20], were procured from Lewis male rats. The limbs were then submerged in 60 mL solution (Steen + with PEG 5% + Trehalose 50 mM + Glycerol varying) in a sealed sterile bag. Starting at 0°C, the limbs were cooled by 1°C every 24h.

### Temperature assessment

Temperature assessment was performed using two techniques. Using thermocouples (TC08 Thermocouple Data Logger, Pico Technology), detailed and continuous temperature measurements were recorded from different regions of the VCA (skin, muscle superficial, muscle deep) by inserting the thermocouples into the tissue and fixing with surgical clips. Temperature was tracked during all phases. For non-invasive temperature tracking of the surface of the VCA a FLIR One Camera (FLIR ONE^®^ Pro – iOS) was used.

### VCA procurement

After induction using isoflurane (5%) inhalation with 100% O_2_, general anesthesia was sustained with inhaled isoflurane (1–3%) and anesthesia depth was confirmed with a toe pinch test. Partial hindlimbs were procured as previously described [19]. Briefly, grafts include the knee joint with 10 mm distal femur and 10 mm proximal femur and tibia, along with thigh muscle groups, the inguinal fat pad and calf muscles as well as the surrounding skin paddle. Femoral vessels were skeletonized and ligated 5 minutes after IV administration of 100IU/mL/kg heparin in the penile dorsal vein. Femoral artery was cannulated with a 24G angio catheter and secured with 6 – 0 nylon suture. Femoral vein was cut after ligation. Immediately after procurement, a pressure-controlled manual flush with 3 mL (200IU) of heparin saline at room temperature was performed. Next, the VCA was subjected to either SNMP to initiate loading phase, or cold stored as described below. VCAs were all procured in 20 minutes or less. Warm ischemia time (WIT) between procurement and connecting to the machine perfusion system was 12 minutes or less.

### Machine perfusion system

Perfusate was circulated using a roller pump system (Masterflex L/S, Vernon Hills, IL) with two separate sets of tubing (Masterflex platinum-cured silicone tubing, L/S 13, Cole-Parmer, Vernon Hills, IL) delivering perfusate into and out of the perfusion reservoir. Temperature was regulated by a water bath (Polystat Cooling/Heating Circulating Bath, Cole-Parmer), set at 21°C or 4°C depending on the phase, through double-jacketed perfusion system components (Radnoti, Covina, CA, USA). Perfusate oxygen concentration was maintained within a close range of 450 mmHg using a 95% O_2_/5% CO_2_ gas cylinder (Airgas, Radnor, PA, USA). Pressure transducer (PT-F, Living Systems Instrumentation, St Albans City, VT) was connected close to the angio catheter (BD Angiocath 24G) in the femoral artery during perfusion.

Vascular resistance (flow and pressure) was monitored continuously; flow rate was manually adjusted to reach a target pressure of 30–35 mmHg. Blood gas and electrolytes in the inflow and outflow perfusate were measured at 30, 60 and 90 minutes during the loading phase and at 60, 90 and 120 minutes during recovery phase using the Siemens RAPIDPoint 500 Blood Gas Analyzer (Siemens Healthineers, Erlangen, Germany). Oxygen consumption was calculated using a modified Fick equation using circuit flow, limb weight, and pre- and post-limb oxygen contents (VO_2_ (mLO_2_/(min*g)) = 0.00314*flow(pO_2_ in – pO_2_ out)/weight). Glucose uptake (mg/h*g) was calculated using the following formula: ((glucose in – glucose out)*flow*60*0.01)/g). Weight (g) was measured after procurement and at the end of each phase. Once the perfusate was warmed to 37°C and oxygenated, pO_2_, pCO_2_, and pH of the solution were verified on the Siemens machine, and NaHCO_3−_ titration was performed to correct for acidosis if needed. The machine perfusion protocol consists of three phases: (1) loading phase; (2) storage phase; (3) recovery phase. For each phase, different solutions were used, which will be described in detail below. For the phases involving perfusion (loading and recovery), modified Steen (Steen+) was used as the base perfusate, which was prepared as described earlier [1]. During the loading phase, Steen + was supplemented with 100 mM 3-O-methyl-glucose (3-OMG) (27760, Chem-Impex, Wood Dale, IL). For the storage phase, CPA composition was based on previous studies and the CPA freezing assessment in vials described above.

### Loading phase

Based on successes in liver and VCA preservation [6, 18], the first 60 minutes of the loading phase consisted of Steen + with 100 mM 3-OMG loading for intracellular cryoprotection. At 60 minutes, temperature is lowered to 4°C and perfusate is switched to CPA solution in 10% increments with a total switching volume of 5 mL, which was based on the average flow rate and time needed to reach a 100% concentration of the CPA solution. At 90 minutes, 4°C is reached, and perfusate is fully switched to the CPA solution. Based on the freezing experiments several CPA solutions are tested. The base consists of HTK with 5g PEG and 50 mM of Trehalose. Based on the CPA freezing results, variable additives are chosen and consist of 5% glycerol (n = 5), 5% EG (n = 4), and 2% glycerol with 15% BSA (n = 3). To assess the effect of stepwise switches, results were compared to previously published results using a supercooling protocol with a flow-controlled single-step CPA flush (glycerol 5% (n = 5); EG 5% (n = 5) flush groups; SCS control group (n = 4)).[18] For the single-step experiments, temperature was lowered to 4°C until 90 minutes was reached. Next, VCA was detached from the system and underwent a single-step flush with 5 mL CPA solution at a flow of 0.5 mL/min.

### Storage phase

VCAs are weighed and submersed in a sterile bag containing 60 mL of 4°C HTK, after which they are sealed, removing the air-liquid interface, and submerged in refrigerant at −4°C to minimize ice nucleating factors such as vibrations [21]. After storage, VCAs are weighed and undergo the same recovery protocol.

### Recovery phase

To avoid the occurrence of ice formation, VCAs were gradually rewarmed to 0°C in a water bath at 37°C for 1 minute and 30 seconds based on the temperature tracking results. Next, perfusion is initiated at 4°C with the CPA solution. Upon connection of the organ, temperature is increased to 21°C and perfusate is switched to Steen + in incremental steps of 10% with a total volume of 5 mL. Recovery with Steen + is continued for 2h. Recovery is performed at 21°C as earlier studies have shown graft viability improvement confirmed by transplantation using this protocol, and have deducted rudimentary transplant criteria based on the perfusion parameters at the end of this phase which are used for reference [1, 5].

### Histology

After recovery phase, 5×5 mm biopsies are taken of skin, muscle and vasculature. Biopsies are fixed in formalin and processed for histopathological examination. Slides are stained with hematoxylin and eosin (H&E). A blinded evaluation by a pathologist is performed for all biopsy samples, and using the muscle injury score [22–24]. For the muscle samples at end of recovery phase, a mean score is calculated for comparison.

### Statistical analysis

CPA freezing probability is examined using Mantel-Cox Assessment. Continuous data is reported as mean and error with range. Perfusion parameters and histology score differences between groups are analyzed using 2-way ANOVA with multiple comparisons or using a mixed-effect analysis when necessary. Outliers are identified using ROUT, Q = 1%. All statistical analyses are performed using Prism 9 for Mac OSX (GraphPad Software, La Jolla, CA). p-Values less than 0.05 are considered to be significant.

## RESULTS

### CPA optimization

Figure 2 displays at which subzero temperature solutions froze. UW showed a lower freezing point than both HTK and Steen+ (Fig. 2A). However, all three solutions froze at lower temperatures than the currently used supercooling temperatures for organs (−2 – −6°C). Remarkably, increasing levels of glycerol did not necessarily suppress the freezing point in all replicates of each group. While freezing started to occur at a lower temperature in the 5% group, the lowest freezing temperatures were found in the 2–4% group. Nonetheless, when taking the probability of freezing into account, the 5% glycerol groups showed the most consistent freezing temperatures amongst all replicates, which results in the most reliable suppression of freezing down to −7°C (Fig. 2B). Interestingly, the 5% glycerol in the supercooling solution showed a larger range of freezing temperatures than HTK by itself with freezing occurring between − 6°C and − 16°C versus at −9°C. Comparing 5% glycerol with 5% EG, a similar range of freezing was found. When compared to the freezing temperatures of the 5% glycerol in the supercooling solution (Fig. 2C), the addition of BSA and 3-OMG caused freezing at slightly higher temperatures compared to 5% glycerol (−5° vs −6°C, respectively). However, the addition of BSA and especially 3-OMG did suppress the lowest freezing temperature. Without the additions, HTK froze at −9°C, but with the additions, at least 50% of all solutions were frozen at −7°C. For the 2% glycerol with BSA solution, freezing occurred at higher temperatures than without BSA (between − 2 and −6 vs −5 and − 16°C). **Figure S1** shows the freezing temperatures of solutions containing partial and whole hindlimbs. These solutions froze between − 6 and − 8°C.

### Thermal tracking

**Figure 3:**
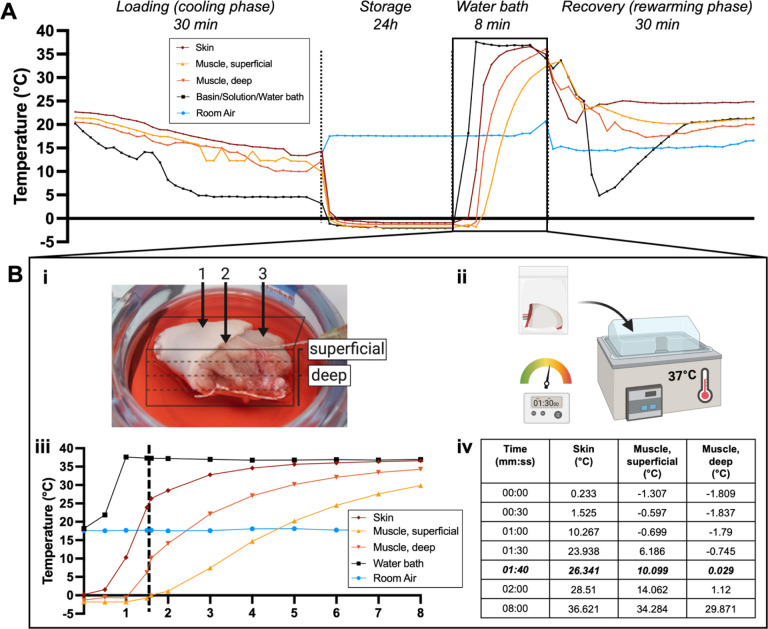
Thermal tracking shows the temperature profile during all stages of the supercooling protocol. (**A**) The temperature of the VCA (15g) was tracked invasively throughout the supercooling protocol. (**B**) Temperature dynamics during rewarming in the water bath are shown in more detail. (**B-i**) Exact thermocouple position in the skin, superficial muscle, and deep muscle, is displayed. (**B-ii**) After 24h supercooling at −4°C the bag was placed in the water bath for 8 min. (**B-iii**) Temperature recording shows that the center of the VCA requires more time to reach 0°C. (**B-iv**) The timetable shows that the VCA core reached 0°C at 1m40s and 29°C at 8 min.

**Figure 4:**
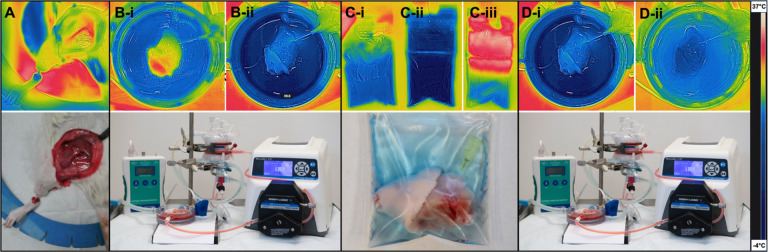
Continuous visual temperature tracking using FLIR imaging. [25] Non-invasive thermal imaging and (**Bottom**) representative clinical images show the VCA at all stages of the supercooling protocol. (**A**) During surgery, the vessels are the warmest structures, while the foot shows cooler temperatures after blood flow has been cut off. (**B**) During the loading phase, the temperature of the VCA is decreased, after which it is added to a (**C-i**) bag with CPAs at 4°C. (**C-ii**) After supercooling, the bag with VCA is at −4°C. (**C-iii**) Here, the VCA is shown in the bag after 1.5 min in the water bath. (**D**) The beginning and (**E**) end of the recovery phase show reheating of the VCA to 21°C.

Figure 3A shows continuous temperature measurement of the VCA throughout the supercooling protocol. During the loading phase, temperature of the VCA lowers to about 10–15°C. While the VCA is inserted in the supercooling bag, little change in temperature occurs (9.99–13.38°C). Once the VCA is placed in the cooler, over the course of 15 minutes for the periphery and 81 minutes for the center, subzero temperatures are reached in the entire graft. Once a temperature equilibrium is reached graft temperature remains stable during the storage time. After storage, when the VCA is placed in a 37°C water bath (Fig. 3B–ii), it reaches 0°C between 30 seconds for the skin and 1 minute and 40 seconds for the center of the graft (Fig. 3Biii–iv). Detailed analysis of the temperature development in the water bath is displayed in Fig. 3B and shows that the core requires the longest time to reheat and that a warming time of 1m30s is sufficient for reaching a temperature of around 0°C. Figure 4 shows representative FLIR images of the VCA, visually and quantitatively substantiating the described temperature profiles of each phase using the 1m30s rewarming time. Supplementary Videos show development of the temperature during the loading phase (1), recovery phase (2) and during a cold flush (3). Here, the distribution of the temperature, i.e. perfusate, throughout the graft can be observed.

### Stepwise switches reduce damage to VCAs

**Figure 5:**
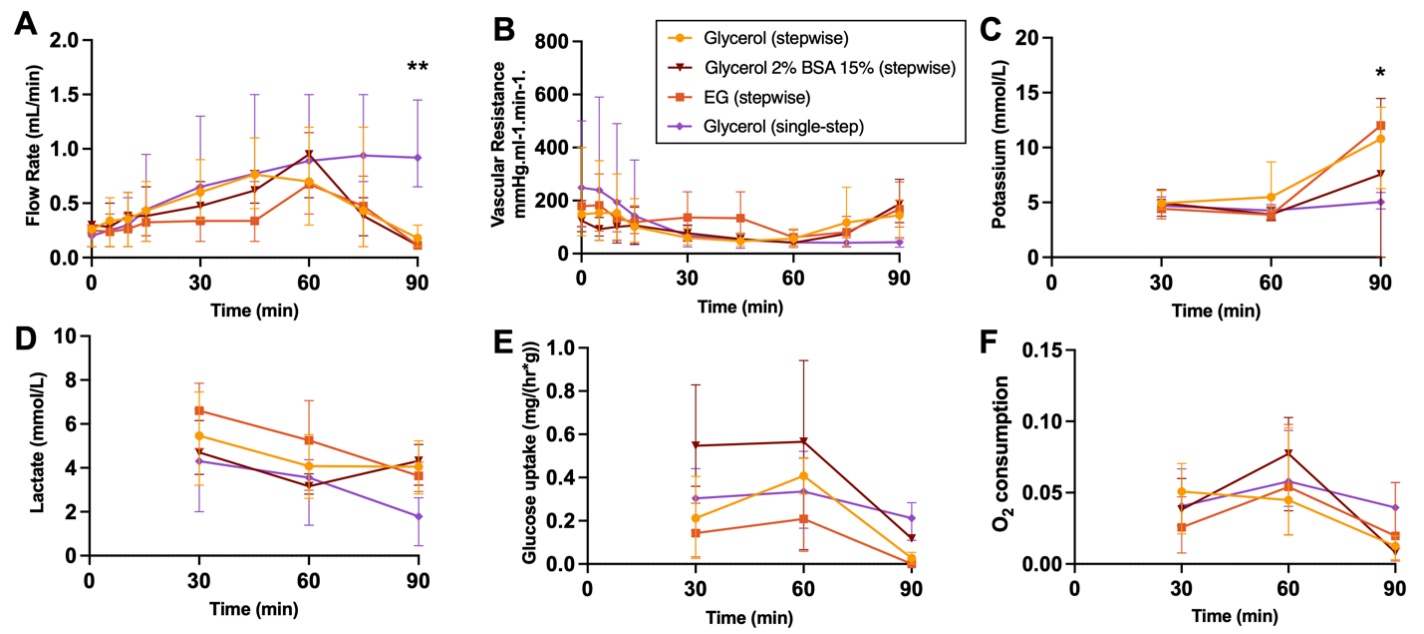
Perfusion parameters during the loading phase. (**A**) Flow rate shows a decrease of flow at 60 min in the stepwise groups due to loading of CPAs as temperature is decreased. (**B**) Arterial resistance also reflects this. (**C**) Potassium is increased at 90 minutes, suggesting the successful loading of CPAs. (**D**) Lactate levels improve over time and are similar between groups. (**E**) Glucose uptake reduces during loading of 3-OMG. (**F**) Oxygen consumption decreases with the lowering of temperature. * p ≤ 0.0332; ** p ≤ 0.0021.

Perfusion parameters of the loading phase are displayed in Fig. 5.VCAs reach a maximum flow of 0.55–1.2 mL/min. VCAs undergoing stepwise loading of CPAs show a decrease in flow at 90 min due to the vasoconstriction occurring upon cooling as well as the increased viscosity of the CPAs, revealed by the lower flow rates and higher resistances in the stepwise group compared to the single-step group, which is not switched to CPAs until after the loading phase. Potassium shows an increase in all stepwise groups at 90 min, which confirms successful loading of the CPAs as they contain high levels of potassium. Lactate levels decrease over time, suggesting washout, with no significant differences between groups. Glucose uptake and oxygen consumption reduce over time in all groups, with lowest levels at 90 min.

**Figure 6:**
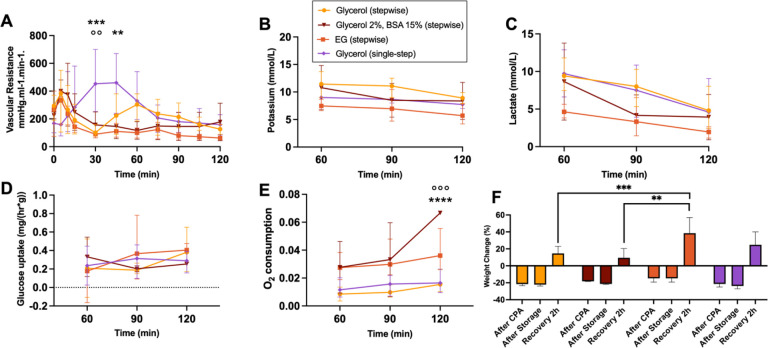
Perfusion parameters during the recovery phase. (**A**) Vascular resistance decreased faster in the stepwise groups (**B**) Potassium and (**C**) lactate level decreased over the course of recovery in all groups. (**D**) Glucose uptake showed an increase in the stepwise Glycerol 5% and EG group, with no significance. (**E**) Oxygen consumption was highest in the stepwise Glycerol 5% BSA15% group at 90 min compared to the single-step group (°) and the stepwise Glycerol 5% group (*). (**F**) Weight changes were highest in the stepwise EG and single-step group. **/°° p ≤ 0.0021; ***/°°° p ≤ 0.0002; **** p ≤ 0.0001

One replicate in the Glycerol 2% BSA 15% group froze overnight, all other replicates across groups remained supercooling until storage was ended. Perfusion parameters of the recovery phase are shown in Fig. 6. Vascular resistance shows a significant increase in the single-step group compared to the stepwise Glycerol 5% at 30 min (p = 0.0008) and 60 min (p = 0.0084), and the stepwise EG group (p = 0.0015) at 30 min. Moreover, the stepwise Glycerol 5% group showed a second increase at 60 min which was not observed in the other stepwise groups. At the end of recovery, vascular resistances were similar between groups. Potassium and lactate levels showed no significant differences between groups, although levels reduced over time in all groups and showed the lowest levels in the stepwise EG group. Glucose uptake increased over time in all groups, with the most drastic increase in the stepwise Glycerol 5% group. Oxygen consumption was highest in the stepwise Glycerol 2% BSA 15% group, with a significant difference reached compared to the single-step group (p = 0.0002) and stepwise Glycerol 5% group (p < 0.0001) at 120 min. While weight loss after loading was lowest in the stepwise EG group, weight gain at the end of recovery was significantly higher compared to other groups, particularly than the stepwise Glycerol 5% group (p = 0.0005) and stepwise Glycerol 2% BSA 15% group (p = 0.0012). Detailed analysis of the stepwise EG group compared to the single-step EG group is shown in **Figure S2**. Histology showed mild to moderate ischemic injury, with no significant differences between the muscle scores of each group.

## DISCUSSION

This study demonstrates a practical method for CPA freezing point assessment. Furthermore, invasive and non-invasive thermal tracking shows the temperature profile of the VCA throughout the supercooling protocol, which led to reduction of the rewarming time and thereby limiting unnecessary WIT. Moreover, in ex vivo experiments we show the benefits of pressure-controlled, stepwise switching between solutions and lower CPA concentrations.

Optimization of CPA freezing points prior to ex vivo studies, allows for assessment of freezing probability, which is especially relevant in an unstable phase such as supercooling. For hearts, zebrafish has been shown to be a useful high-throughput model for organ preservation research.[9] Especially useful here is the ability to assess heart function in the zebrafish. For VCAs such a model does not exist. However, ice nucleators seem to have been the main challenge for VCAs, considering VCAs inherently contain ice nucleators such as hair, fat, bone, and nails which are in contact with the storage solution during the storage phase. Therefore, assessing freezing points with the aimed tissues and volumes is of interest and can provide more precise prediction of the solution’s stability. In this study, this was demonstrated by the higher freezing probability of the Glycerol 2% BSA 15%, which was indeed the only group with one replicate that froze. As such, this step of performing CPA optimization prior to animal studies can reduce the number of live animals needed as no functional analysis is necessary until stable CPA combinations are established. In terms of relevance to the clinic, being aware of which ingredients influence the solution’s stability at subzero temperatures has relevance, as it will inform decision-making on which essential ingredients to include and at which temperatures to store the organs. The lower the temperature, the lower the tissues metabolism and this degradation, however, if the risk of freezing is too high, the risks will not outweigh the benefits. To increase efficiency further, future studies could focus on the development of a functional high-throughput model specifically for VCAs, such as an in vitro cell model or microfluidic device[26], which can be used of CPA optimization prior to performing ex vivo studies.

Our thermal tracking methodologies provided insights into the temperature profiles within VCAs during supercooling. Even though thermocouples are invasive, they provide valuable insight into the temperature profile and (in)homogeneity throughout the supercooling phases. By establishing a heating time of 1.5 minutes to reach 0°C, we provide a concrete, evidence-based recommendation to minimize WIT [5] which can also be established for larger organ models (e.g. human upper extremity) using the same technique. Granted, the fast rewarming time in this study is due to the small size of the tissue, this tissue size is comparable to the size of a human digit. Larger specimens, such as human arms would be expected to require longer rewarming times, in the realm of tens of minutes, which can be tested more precisely using the methods shown in this study. The FLIR imaging technique offers a non-invasive method for assessing temperature changes, thereby balancing between detail and tissue integrity. Use of the technique during temperature changes is not unlike dynamic infrared thermography (DIRT), used for the detection of perforator mapping and providing information on arterial and venous hemodynamics by the use of air flow cooling [27, 28]. On the downside, this technique only allows for assessment of superficial tissue due to limited penetration depth. With this in mind, there could be a role for FLIR during machine perfusion as a non-invasive method for assessing perfusion and thermal dynamics in the superficial regions of the tissue.

Perhaps most notably, the study’s ex vivo experiments, involving stepwise CPA loading versus traditional single-step protocols, reveal an advantage in controlled, gradual CPA introduction. During the loading phase, no differences are seen, apart from those that are direct consequences of the stepwise loading during the cooling phase, such as lowering of the flow due to increased viscosity and decreased temperature, and increase of potassium due to high potassium concentrations in HTK at 90 min. During the recovery phase, improved arterial resistance and lower weight change was observed in stepwise CPA loading compared to the single-step group. Possible explanations are a reduction in endothelial damage and osmotic shock, which would explain why the 2% Glycerol 15% BSA group shows the most improved recovery with higher oxygen consumption and lower weight gain than the other groups. These findings challenge the existing single-step protocol and make provision for more refined approaches in VCA preservation, with the aim of protecting the organ against damage.

Limitations of this study include challenges posed by small animal ex vivo models, such as the low flow which can influence replicability. However, with complex experiments such as the ex vivo experiments performed in this study, breaking down some of the variables in simpler models such as freezing assessment in vials and determining heating dynamics, increases the reliability of results, and simultaneously increases the replicability. Furthermore, upon translation, some of the complicating variables in small animal models, such as low flow, are not expected to be an issue in large animal models [7]. Conversely, large models will bring their own challenges with increasing volumes. Further improvements to the supercooling protocol would allow for the transplantation of supercooled grafts, thereby providing additional insights into the postoperative development of the grafts and their differences compared to VCAs that underwent traditional SCS. Major hurdles to overcome are to control the substantial weight changes and the development of an optimal storage solution that fulfills the needs of VCAs. Of special interest would be a deeper understanding of the capillary network and endothelial function in VCAs. It is known that the endothelium and number of capillaries are organ-specific and are present in high numbers in muscle tissue. The endothelium contains tight junctions, in contrast to, for example, liver endothelium, which influences the leakage permitted past this barrier. Future studies that elucidate the endothelial function in VCAs and how this can be modulated to improve preservation outcomes, will be of great interest to the preservation field as well as directly relevant to clinical practice. In reconstructive surgery, recovery of tissue such as digits or even entire extremities, is severely limited by the maximum preservation time of 6 hours. Not only for military personnel in low-resource settings but also in daily clinical practice, 6 hours to reach a specialized hospital, create a personalized reconstructive plan, and perform the surgery is logistically challenging. Even when VCAs are successfully replanted, during follow-up, edema remains a challenge, delaying wound healing or even causing further damage by compressing critical structures such as arteries.

In conclusion, our study broadens the existing knowledge in the field of organ preservation by introducing new considerations such as CPA optimization prior to ex vivo and in vivo studies, thermal tracking, and use of stepwise switches between preservation solutions, taking osmotic variations into account. By highlighting the effectiveness of novel preservation techniques and methodologies, this research sets a new direction for enhancing VCA preservation, providing methodological guidance to effectively improve outcomes, which can be applied when upscaling to large animal models and to transplantation outcomes.

## Data Availability

All data generated and analyzed during this study have been included in this manuscript and its Supplementary Information file unless stated otherwise. All raw data can be provided upon request from the corresponding author.
